# First Food Policy and Law Scan: How Tribes in the Bemidji Area Are Applying Policy and Systems Approaches to Support Breastfeeding

**DOI:** 10.5888/pcd18.200460

**Published:** 2021-07-22

**Authors:** Julie Ralston Aoki, Meghan A. Porter

**Affiliations:** 1 Public Health Law Center, St. Paul, Minnesota; 2 Great Lakes Inter-Tribal Epidemiology Center, Great Lakes Inter-Tribal Council, Inc, Lac du Flambeau, Wisconsin

## Abstract

The objective of the First Food Policy and Law Scan was to identify breastfeeding policies adopted by federally recognized Tribes in the Indian Health Service’s Bemidji Area to understand how breastfeeding is supported through policy. In 2018–2019, we invited all federally recognized Tribes in the Bemidji Area (Michigan, Minnesota, and Wisconsin) to share information on policies in 6 settings (eg, government, casinos). Tribal contacts shared 61 policies from 31 Tribes. We analyzed the policies for 17 features. The project demonstrated that one way the Bemidji Area Tribal Nations are addressing chronic diseases is by applying policy to support breastfeeding.

**Summary**

**What is already known on this topic?**

Breastfeeding supports improvements in health outcomes across several chronic disease areas and is a traditional American Indian/Alaska Native practice. Colonization, genocide, and related trauma have disrupted and undermined uptake and maintenance of this healthy practice for many American Indian/Alaska Native families.

**What is added by this report?**

This project highlights how Tribal governments in the Indian Health Service’s Bemidji Area are exercising sovereignty and using law, policy, and systems approaches to support breastfeeding across their communities, including sometimes in ways that surpass US law protections.

**What are the implications for public health practice?**

Tribes have many policy approaches to draw upon to support and protect breastfeeding. Tribal public health leaders, breastfeeding advocates, and community members can draw upon this research to better understand the scope of policies available to implement, and to advocate for culturally tailored policy components.

## Objective

Breast milk is a traditional, Indigenous first food (1,2) that promotes health and prevents chronic disease among infants and mothers (3). The objective of our study, the First Food Policy and Law Scan, was to identify breastfeeding policies adopted by federally recognized Tribes in the Bemidji Area of the Indian Health Service (Michigan, Minnesota, and Wisconsin). We hypothesized that Tribes are exercising sovereignty to support breastfeeding through policy. We also aimed to facilitate the sharing of information about Tribal breastfeeding policies across Tribal communities to promote awareness of how policy can support first food practices and good health for infants, women, and families across the lifespan.

## Methods

The cross-sectional, descriptive legal epidemiology project collected and analyzed information about breastfeeding policies from federally recognized Tribes in the Bemidji Area. We gathered policies from entities in 6 settings: government (administrative policies, Tribal law), health care (Tribally operated and Indian Health Service [IHS] facilities), casinos, early childcare and education (ECE), Tribally operated Bureau of Indian Education schools, and Tribal colleges. We included the IHS facilities because of their importance to the Tribes they serve. We classified policies as formal or informal. “Formal” policies were adopted by decision makers (eg, Tribal Council, ECE director) *and* in writing (eg, statute, staff handbook). “Informal” policies were those that contacts described as policies but were not documented in writing.

We extended invitations to participate in the project via both letters and emails sent to Tribal leaders, legal counsel, and health directors, asking them for access to information on policies as well as referrals to contacts in relevant settings. Participation was optional and was demonstrated by sharing of policies. Some Tribes publish laws online; for these Tribes, we notified legal counsel that we would collect relevant laws from online sources, but they could opt out if desired. Invitations explained that we would write an aggregate report but would not include identifiable information without permission, to respect Tribal sovereignty.

We collected information on policies from online legal databases and Tribal websites, and by email and telephone, from June 1, 2018, through August 31, 2019. We asked contacts to provide written and unwritten breastfeeding policies. We then sorted policies by Tribe, setting, and formal/informal status. We developed and used a coding protocol consisting of 17 features (Box). For the aggregate report, we asked contacts to review relevant sections of the draft for accuracy and to confirm permission to identify policies. We also developed confidential briefs that summarized findings for participating Tribes.

**Box. 17 Coding items used to analyze breastfeeding policy information from federally recognized Tribes in the Bemidji Area**

1. Policy focus

• supporting milk expression (ie, pumping)

• allowing nursing babies on-site

• both supporting milk expression and allowing nursing babies on-site

• right to breastfeed

• breast milk use or storage

• Other/unclear (eg, establishing a breastfeeding peer counselor position, exempting nursing women from indecent exposure laws)

2. Purpose/intent of language

3. Type of space/facilities

4. Break time guidelines (frequency, paid/unpaid)

5. Infant age limits

6. Expressed milk storage provisions

7. Access to sink or other cleaning facilities

8. Standards for implementation

9. Obligations of covered persons (eg, employees)

10. Enforcement provisions

11. Exemptions

12. Anti-discrimination language

13. Infant feeding practices

14. Support services (eg, educational programs, lactation counselor)

15. References to Tribal culture (including language)

16. Evaluation

17. Other

We tabulated results according to policy type (informal, formal), the feature of policy focus (ie, the policy’s main goal/topic), and other selected features. We highlighted certain policy features in our analysis because of their salience in supporting breastfeeding in Tribal communities (eg, references to Tribe’s culture or language) (4,5) or because they exceeded federal protections.

## Results

Contacts in 31 of 34 federally recognized Tribes contributed information on 61 policies, including 31 formal policies and 30 informal policies. One Tribe responded they had no policies to share and 2 Tribes did not participate.

Casinos and health care settings shared the most policies, with each setting yielding 16 policies (32 policies total) followed closely by Tribal government settings (n = 13 policies). The most common policy foci were support of milk expression, and the support of milk expression combined with allowing the nursing of babies on-site (Table). The least common was allowing the nursing of babies on-site without also addressing milk expression. Coders were able to assign all but 2 policies into 1 focus category; the exceptions were 2 ECE policies that we determined had a focus both on nursing baby on-site/milk expression and milk use/storage.

**Table:** Number of Breastfeeding Policies Among Federally Recognized Tribes in Michigan, Minnesota, and Wisconsin, Grouped by State and Setting, June 1, 2018–August 30, 2019

State/Setting	Policy Type			Policy Focus					Other Selected Features			
	Informal	Formal	Totals	Allowing Nursing Baby On-Site	Supporting Milk Expression	Nursing Baby On-Site and Milk Expression	Right to Breastfeed	Breast Milk Use, Storage, or Other Focus	Reference to Culture or Language	Paid Breaks	Baby Age Limit <12 Mo	Evaluation
**Michigan**												
Health	1	4	5	0	0	3	0	2	1	0	1	0
Gaming	2	1	3	0	1	1	0	1	1	1	1	0
Education	0	2	2	0	1	1	0	0	0	0	1	0
Childcare/early childcare and education	1	2	3	0	0	2	0	1	0	0	0	0
Code/government	0	3	3	0	0	1	1	1	1	0	0	0
Subtotal	4	12	16	0	2	8	1	5	3	1	3	0
**Minnesota**												
Health	1	4	5	0	0	4	0	1	0	1	1	1
Gaming	8	0	8	0	8	0	0	0	0	1	0	0
Education	2	1	3	0	1	2	0	0	0	0	0	0
Childcare/early childcare and education	1	3	4	0	0	3	0	3	0	0	0	0
Code/government	1	0	1	0	1	0	0	0	0	0	0	0
Subtotal	13	8	21	0	10	9	0	4	0	2	1	1
**Wisconsin**												
Health	2	4	6	0	3	2	0	1	1			
Gaming	5	0	5	0	3	1	0	1	0	0	1	0
Education	1	0	1	0	1	0	0	0	0	0	0	0
Childcare/early childcare and education	2	1	3	1	0	0	0	2	0	0	0	0
Code/government	2	7	9	0	4	1	3	1	4	1	0	1
Subtotal	12	12	24	1	11	4	3	5	5	1	1	1
Grand totals	29	32	61	1	23	21	4	14	8	4	5	2

Formal policies varied in complexity. Gaming facility policies typically addressed supports for employees. Education setting policies addressed supports for staff members and sometimes for students and visitors. Health setting policies typically addressed supports for patients, staff members, and sometimes community members (eg, breast pump loan policy). ECE policies typically addressed supports for parents and providing breastmilk to babies in the program. Government policies included employee support policies, right-to-breastfeed resolutions, laws prohibiting discrimination against pregnant and nursing mothers, and a law clarifying that breastfeeding did not violate indecent exposure laws.

Informal policies typically focused on providing private lactation spaces and related facilities (eg, refrigerators for milk storage). Some informal policies reflected a community-wide approach, with contacts describing multiple lactation rooms available in several settings or multiple activities to support breastfeeding (eg, educational campaigns, providing comfortable/private spaces at powwows and other community events).

## Discussion

No studies have systematically documented how Tribes support breastfeeding via policy. We found that Bemidji Area Tribes apply sovereignty to support breastfeeding in multiple ways. The number of employee policies supporting breastfeeding overall (and not only milk expression) was noteworthy; non-Tribal entities predominantly focus on milk expression policies (6). Additionally, Tribal health departments were key partners in several policy efforts. For example, in some Tribes, health departments promulgated administrative policies covering all Tribal employees or provided lactation spaces for casino employees or at community events. These examples indicate that cross-agency collaborations can be important for supporting breastfeeding in a community. Given the cultural significance of first food practices, we expected many references to Tribal culture or language but relatively few policies included them. Incorporating Tribal culture and language into breastfeeding policies could be an area for Tribal policy makers and health advocates to explore.

Our analysis has several limitations. First, policies were likely underreported because the correct contact person may not have been identified or was busy, or for other reasons. Some contacts reported policies in development but not completed. Second, policy counts have limited utility. One comprehensive law that covers the Tribe’s entire jurisdiction can be powerful, despite being only one policy. Additionally, the number of entities and agencies varies across Tribes. For example, some Tribes have 1 casino or 1 ECE program; others have several where all may follow the same policy, or each facility may have its own. Also, there are relatively few Tribally operated Bureau of Indian Education schools and Tribal colleges. Third, policy detail and length varied greatly; information provided about informal policies was subjective and often sparse. Finally, we documented the existence of policies; implementation of policies also warrants research.

As Tribes strive to prevent chronic disease, leveraging policy is a method already used by many settings and communities to support first food feeding practices.
